# Labyrinthitis Complicating Acute Otitis Media in an 18-Month-Old Infant

**DOI:** 10.7759/cureus.88823

**Published:** 2025-07-26

**Authors:** Yahya Boualam, Achraf Sbai, Drissia Benfadil, Azzedine Lachkar, Fahd El Ayoubi El Idrissi

**Affiliations:** 1 Department of Otorhinolaryngology, Mohammed VI University Hospital Center, Oujda, MAR; 2 Department of Otorhinolaryngology, Faculty of Medicine and Pharmacy, Mohammed First University, Oujda, MAR

**Keywords:** bilateral congenital cholesteatoma, computed tomography (ct), ossicular chain lysis, ossiculoplasty, otologic surgery, otoscopy

## Abstract

Labyrinthitis is a peripheral vestibular disorder characterized by the sudden onset of vertigo and imbalance. It typically affects adults and can significantly impair daily functioning. Diagnosis relies on clinical evaluation and the exclusion of central causes. Early management is crucial to reduce the duration of symptoms and prevent complications. Long-term outcomes vary, with some individuals experiencing persistent vestibular dysfunction. This report presents a case of an 18-month-old infant diagnosed with labyrinthitis of the right ear secondary to acute otitis media.

## Introduction

Labyrinthitis is a condition characterized by inflammation of the membranous labyrinth, typically resulting from a viral or bacterial infection, or as a manifestation of an underlying systemic disease [1]. Clinically, it presents with vertigo, nausea, vomiting, and may be accompanied by tinnitus or hearing loss. In some cases, labyrinthitis may develop as a complication of either acute or chronic otitis media [2,3]. Treatment depends on the underlying cause and may include antibiotics, corticosteroids, or supportive care measures. The condition often progresses toward full recovery; however, some patients may experience long-term sequelae, particularly balance disorders and hearing impairment [1].

## Case presentation

An 18-month-old infant was admitted to the pediatric emergency department with fever, irritability, gait disturbances, and an inability to stand. According to the parents, a consultation with a general practitioner had taken place one week earlier due to persistent crying and fever. At that time, a diagnosis of non-suppurative acute otitis media of the right ear was made, and the infant was prescribed symptomatic treatment consisting of paracetamol and nasal irrigation.

Over the following days, the child’s condition worsened. Two days prior to admission, new symptoms developed, including gait instability and a complete inability to stand, accompanied by persistent fever and increased irritability, prompting the parents to seek emergency medical attention.

On physical examination, the infant exhibited horizontal nystagmus beating to the left. Otoscopic examination revealed a mildly inflamed tympanic membrane on the right side.

Laboratory investigations showed a slightly elevated C-reactive protein (CRP) level with leukocytosis at 19,540/mm3, predominantly lymphocytic (13,000/mm3). The remainder of the biological workup was unremarkable.

**Table 1:** CRP and white blood cell values in our patient compared to normal reference ranges.

Parameter	Result	Normal Range (18 months)
CRP (C-reactive protein)	6 mg/L	< 5 mg/L
White blood cells (WBC)	19,540 /mm³	6,000 – 17,500 /mm³

**Figure 1:**
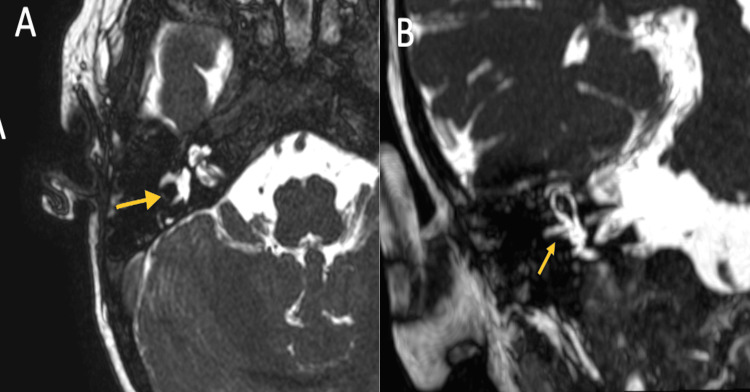
MRI 3D FIESTA sequence on the right side (A: axial plane, B: coronal plane): the hyperintense signal of the lateral semicircular canal ring (yellow arrow) is discontinuous. FIESTA: Fast imaging employing steady-state acquisition

A cerebral magnetic resonance imaging (MRI) was performed, revealing opacification of the mastoid air cells and a signal drop in the right lateral semicircular canal (Figure 1), findings consistent with labyrinthitis. No intracranial abnormalities were identified. 

**Figure 2:**
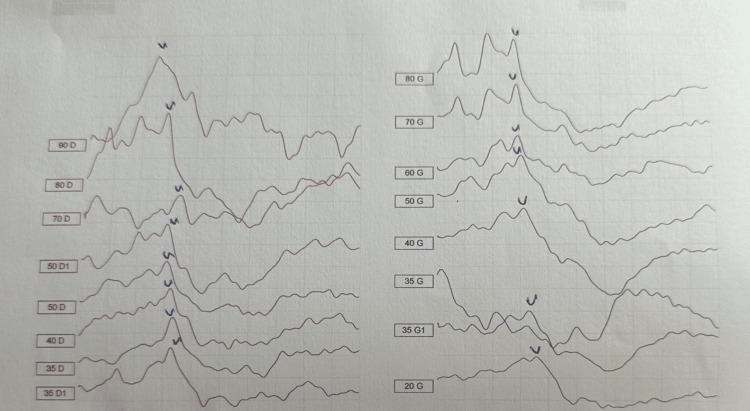
AEPs showing mild hearing loss on the right side with an auditory threshold of 35 dB. AEP: Auditory evoked potential

Auditory evoked potentials were performed 48 hours after hospitalization (Figure 2), revealing mild right-sided hypoacusis with an auditory threshold of 35 dB, while hearing on the left side was normal.

The infant was admitted for inpatient care with multidisciplinary management by pediatricians and otolaryngologists. Intravenous antibiotic therapy was initiated using amoxicillin-clavulanic acid at a dose of 80 mg/kg/day, administered in three divided doses, in combination with corticosteroids at a dose of 2 mg/kg/day for eight days, and antipyretics (paracetamol at 60 mg/kg/day divided into four doses every six hours).

The clinical course was marked by resolution of fever within 48 hours, normalization of laboratory parameters by day 4, and recovery of balance, characterized by the disappearance of nystagmus and gait disturbances (independent walking, ability to stand up without support, presence of postural reflexes, and stair climbing with assistance) by day 8.

After completing 10 days of intravenous antibiotic therapy and eight days of corticosteroid treatment, the patient was discharged with the approval of the pediatric team. Follow-up was scheduled in both ENT and pediatric outpatient clinics every two weeks during the first month, then monthly for the following three months, with brainstem auditory evoked potentials (BAEPs) testing planned at six months.

## Discussion

Tympanogenic labyrinthitis is a rare complication of otitis media and may be either serous or suppurative [1,4]. It is typically caused by a viral or bacterial infection; however, it may also represent a manifestation of systemic disease, including human immunodeficiency virus (HIV) infection [5,6]. The annual incidence of labyrinthitis is approximately 0.1%, with a prevalence estimated at 13%, primarily affecting underserved populations in both developing and developed countries [3,4]. Viral labyrinthitis is the most common form and is usually secondary to an upper respiratory tract infection [5]. Suppurative bacterial labyrinthitis, most commonly arising as a complication of bacterial meningitis, remains the leading cause of hearing loss in children under two years of age. However, in the post-antibiotic era, its occurrence has become exceedingly rare. Otogenic suppurative labyrinthitis, which may occur at any age, is typically associated with cholesteatoma or results from chronic, untreated otitis media [7,8].

Severe vertigo, nausea, and vomiting are the classic clinical manifestations of labyrinthitis [9]. The acute vertiginous episode rarely lasts more than 72 hours; however, balance disturbances, mild residual vertigo, as well as hearing impairment and tinnitus, may persist for several weeks [7]. On examination, patients typically present with balance and gait disturbances, as observed in our patient. Nystagmus is a hallmark finding, with the fast phase beating toward the healthy ear. Otoscopic evaluation may reveal the underlying cause of the labyrinthitis, such as otitis media or cholesteatoma [10].

Audiometry is essential for assessing the degree of sensorineural hearing loss. Advanced vestibular assessments, such as vestibular evoked myogenic potentials (VEMPs), electronystagmography (ENG), and rotary chair testing, are generally not recommended during the acute phase but may be valuable in assessing long-term vestibular compensation and identifying residual functional impairment.

Laboratory investigations are guided by the patient’s clinical presentation and differential diagnoses. Initial evaluation should include inflammatory markers, such as complete blood count (CBC) and C-reactive protein (CRP), to assess for inflammation. In patients presenting with vomiting, serum urea and electrolyte levels should be assessed, and any deficiencies corrected. Serological testing for HIV and syphilis should be conducted, and an autoimmune workup considered if systemic signs are present. A lumbar puncture is indicated in patients with clinical signs suggestive of meningitis.

Radiologically, magnetic resonance imaging (MRI) is the modality of choice for initial evaluation, as it provides diagnostic confirmation and helps exclude differential diagnoses [11-13]. In our case, MRI demonstrated interruption of the hyperintense signal in the right lateral semicircular canal on 3D FIESTA sequences.

The treatment of labyrinthitis is both etiologic and symptomatic. In cases secondary to otitis media, antibiotic therapy should be initiated, with or without drainage, if middle ear effusion is present. Benzodiazepines and antihistamines may be used during the first 72 hours to alleviate vertigo; however, these should be prescribed for short durations only, as prolonged use may hinder vestibular compensation [1,14].

Corticosteroids may be used as adjunctive therapy. Steroid treatment has been associated with a statistically significant reduction in the incidence of subsequent hearing loss, as reported in several studies. Some authors even advocate for its systematic administration [15], a practice adopted by our team.

The clinical course of labyrinthitis is typically favorable, with resolution of symptoms occurring within 10 days to one month following treatment with antibiotics, particularly amoxicillin-clavulanic acid combined with corticosteroids and vestibular rehabilitation. Nevertheless, some patients may experience persistent sequelae beyond this period [16].

## Conclusions

Labyrinthitis secondary to otitis media remains a rare condition, with vertigo and balance disturbances as its primary clinical manifestations. Diagnosis relies on both clinical evaluation and imaging, particularly MRI. Management is based on a combination of antibiotic therapy, corticosteroids, and vestibular rehabilitation. Although most patients experience full recovery, some may experience persistent deficits lasting several weeks.
